# Relationship Between Precipitation Extremes and Convective Organization Inferred From Satellite Observations

**DOI:** 10.1029/2019GL086927

**Published:** 2020-05-06

**Authors:** Addisu Gezahegn Semie, Sandrine Bony

**Affiliations:** ^1^ Laboratoire de Meteorologie Dynamique (LMD/IPSL), Sorbonne University, CNRS Paris France; ^2^ Computational Data Science Program Addis Ababa University Addis Ababa Ethiopia

**Keywords:** precipitation extremes, convective organization, tropical convection, satellite observations

## Abstract

Convective organization has the potential to impact the strength of precipitation extremes, but numerical models disagree about this influence. This study uses satellite observations to investigate the link between the mesoscale organization of deep convection and precipitation extremes in the Tropics. Extremes in domain‐averaged precipitation are found mostly over the western Pacific and Indian Ocean warm pools, and they primarily depend on the number of deep convective entities within the domain. On the other hand, extremes in local precipitation are found primarily over land, and they increase with the degree of convective organization. Therefore, this observational study shows evidence for a modulation of the strength of tropical precipitation extremes by the spatial organization of deep convection, especially over land.

## Key Points

- The link between tropical precipitation extremes and the mesoscale organization of deep convection is investigated using satellite data
- The strength of local precipitation extremes exhibits a strong dependence on convective organization
- Local precipitation extremes primarily result from changes in the fractional area of heavy precipitation

## 1 Introduction

Organized deep convection is ubiquitous in the Tropics and it occurs over a wide range of spatial and temporal scales. Mesoscale organization and large cloud clusters, that occur on scales of hundred to thousand kilometers (Houze, 2014; Lilly & Gal‐Chen, 2013), include short‐lived aggregates of thunderstorms, well‐organized squall lines, and long‐lived tropical storms and hurricanes. Most extreme events such as heavy rain, flash floods, and severe weather are associated with organized convective systems (Mathon et al., 2002; Nesbitt et al., 2006). When a cloud develops as part of a convective cluster, it tends to be embedded in a moister environment than if it is isolated. Being less subject to the entrainment of dry environmental air, convective updrafts can be more intense, and the falling rain being less subject to reevaporation, the precipitation efficiency is enhanced. Both processes can affect the dynamics of precipitation extremes and might be responsible for cases when the increase of precipitation extremes with warming exceeds the Clausius‐Clapeyron scaling (O'Gorman, 2015).

During the last decade, many modeling studies have investigated the physical mechanisms and the impact of convective aggregation on climate (Bretherton et al., 2005; Coppin & Bony, 2015; Muller & Held, 2012; Wing & Emanuel, 2014), see Wing et al. (2017) for a review. A study using a climate model to simulate radiative‐convective equilibrium (RCE) (Bao et al., 2017; Pendergrass et al., 2016) has found a large increase in extreme precipitation when convection transitions from disorganized to organized states. However, climate models do not represent the mesoscale organization of convection explicitly (Rossow et al., 2013). An idealized simulation conducted with a convection‐resolving model by Singleton and Toumi (2013) also found a significant increase in instantaneous and accumulated (up to 1 hr) precipitation extremes when convection is more strongly organized. In contrast, RCE simulations performed with another mesoscale model found that convective self‐aggregation had a negligible impact on extremes of instantaneous precipitation (Bao & Sherwood, 2019). Therefore, the role that the mesoscale organization of convection might play in precipitation extremes remains uncertain.

In this paper, we use satellite observations to investigate the relationship between mesoscale convective aggregation and extreme precipitation in the Tropics. First, we present our methodology for characterizing the spatial distribution of deep convection and precipitation extremes (section 2). In section 3, we show how extremes in local and domain scale precipitation behave with different degrees of convective organization. Our results are summarized and discussed in section 4.

## 2 Data and Method

Following previous studies of convective aggregation using observations (Stein et al., 2017; Tobin et al., 2012) or cloud‐resolving models (Tompkins & Semie, 2017), we characterize the mesoscale organization of deep convection in longitude‐latitude domains of 10° × 10°.

Within the 20°S to 20°N area, we consider every 1° a moving box of 10° × 10°, thus totaling 14,400 (360 × 40) 10° × 10° domains within the tropical belt.

### 2.1 Characterization of the Precipitation Field

The precipitation data are derived from the version 6 of the Tropical Rainfall Measuring Mission (TRMM) 3B42 product for the period 1998 to 2010 (Huffman et al., 2007). It is a Multisatellite Precipitation Analysis that provides a calibration‐based sequential scheme for combining precipitation estimates from multiple satellites, as well as gauge analyses. It is available at the 3‐hourly time scale with a spatial resolution of 0.25° × 0.25°. It has been shown to reproduce the surface observation‐based histogram of precipitation and to reasonably detect large daily events (Huffman et al., 2010).

The dataset includes undefined data. To ensure a good homogeneity of the data among all 10° × 10° domains within 20°S to 20°N at any given time, we exclude from our analysis all the domains for which more than 1% of their area is covered by undefined precipitation data. When considering all 3‐hourly data over the period 1998–2010, applying this quality criteria reduces the total number of 10° × 10° domains considered in the analysis by about 27% (from 546,868,800 to 397,268,984).

**Figure 1:**
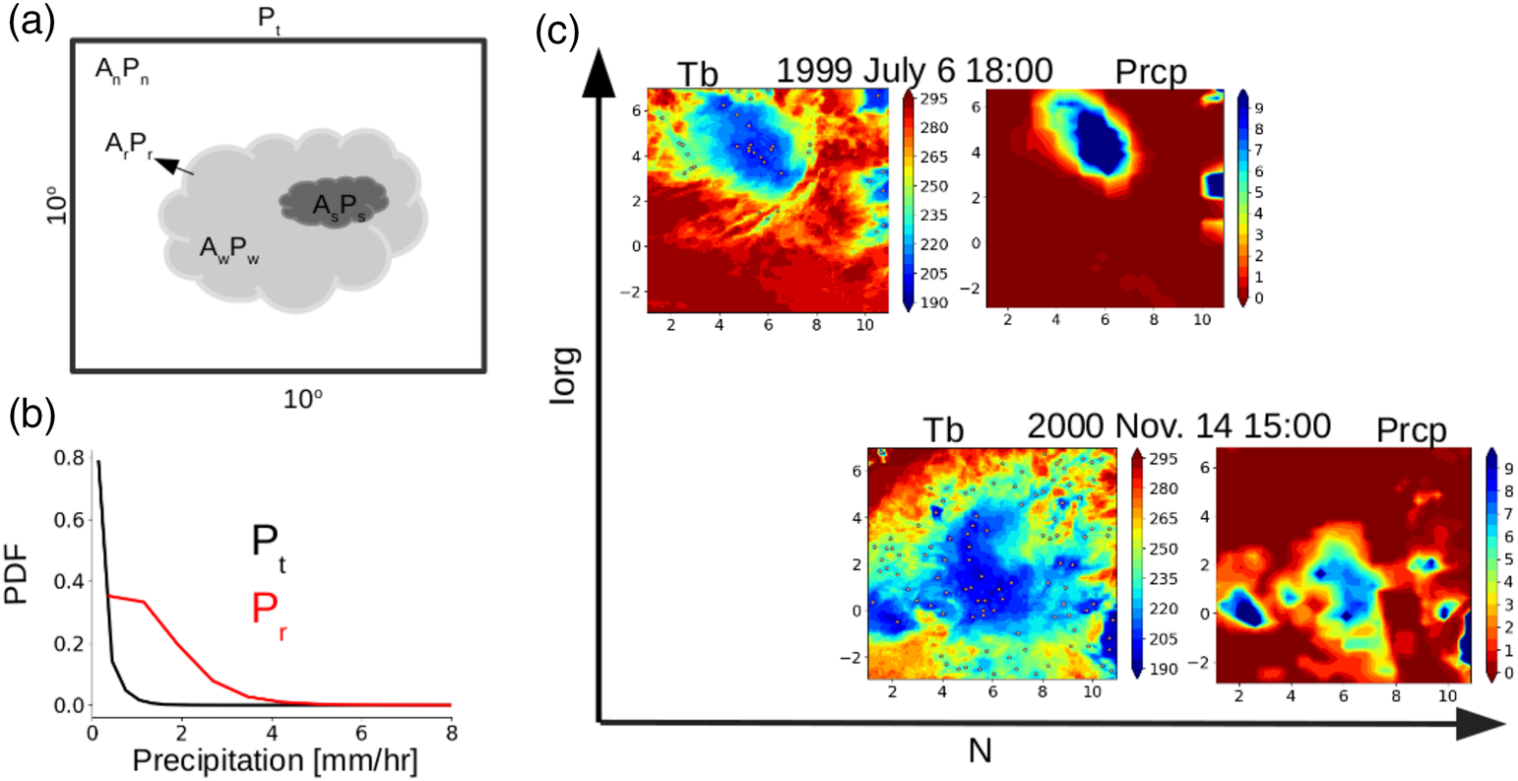
(a) Schematic of precipitation in 10° × 10° domain. Domain mean precipitation *P*_*t*_ consists of nonprecipitating (*P*_*n*_) and precipitating (*P*_*r*_) areas with the corresponding area coverages *A*_*n*_ and *A*_*r*_. The precipitating region *P*_*r*_ is further partitioned into strongly (dark gray) and weakly (light gray) precipitating areas (*A*_*s*_ and *A*_*w*_). (b) Probability density function of *P*_*t*_ and *P*_*r*_ obtained for all 10° × 10° domains. (c) Examples of snapshots of precipitation (in mm hr^−1^) from the TRMM 3B42 dataset and of the infrared brightness temperature (*T*_*b*_ in K) from the GridSat dataset over a given 10° × 10° longitude‐latitude domain at two different dates; the 1999 snapshot is associated with a higher organization index *I*_org_ and a lower N than the 2000 snapshot. Points indicate the deep convective centriods identified through local minimum method.

Then, we characterize the precipitation field within each 10° × 10° domain as represented schematically in Figure 1a: we distinguish precipitating (*A*_*r*_) and non‐precipitating (*A*_*n*_=1−*A*_*r*_) fractional areas (normalized by the 10° × 10° area), and the precipitating area is further decomposed into weakly (*A*_*w*_) and strongly (*A*_*s*_) precipitating areas, whose ratio relative to *A*_*r*_ is referred to as *a*_*w*_ and *a*_*s*_. Each of these areas is characterized by a precipitation intensity (*P*_*r*_, *P*_*w*_, and *P*_*s*_, respectively), so that the domain‐averaged precipitation (*P*_*t*_) can be expressed as

(1) $$\begin{array}{ccc} P_{t} & =A_{r}P_{r}\;\;\;\;\text{with} & \\ P_{r} & =a_{w}P_{w}+a_{s}P_{s}\;\;\;\;\text{with}\\ A_{r} & =A_{w}+A_{s},a_{w}=\frac{A_{w}}{A_{r}}\;\;\text{and}\;\;a_{s}=\frac{A_{s}}{A_{r}} \end{array}$$

The distinction between weakly and strongly precipitating areas is done by considering a threshold value of 2 mm hr^−1^. To test the robustness of our results to this threshold, we also considered alternative values (0.5, 1, 1.5, and 2.5 mm hr^−1^); similar results are obtained for all threshold values greater than 1 mm hr^−1^.

Each 10° × 10° domain includes 40 × 40 (1,600) TRMM pixels of 0.25° × 0.25°. *P*_*r*_ is defined as the average value of pixels having values greater than 0 mm hr^−1^, while *P*_*t*_ is defined as the average value of all grid points (both precipitating and non‐precipitating) within the 10° × 10° area. In the following, we will refer to *P*_*t*_ and *P*_*r*_ as domain‐scale and local‐scale precipitation, respectively. Figure 1b shows the probability density function of the *P*_*t*_ and *P*_*r*_ values obtained for all the domains satisfying the quality criteria discussed above. Over most of the domains, *P*_*t*_ and *P*_*r*_ are lower than 1 mm hr^−1^ and 4 mm hr^−1^, respectively (Figure 1b).

### 2.2 Precipitation Extremes

All precipitation diagnostics *P*_*t*_ and *P*_*r*_ are calculated for each 10° × 10° domain, and then we aggregate all values in space (20°S to 20°N) and time (1998 to 2010). Precipitation extremes are then defined as the 99th percentile value of this ensemble. The 99th percentile values of *P*_*t*_ and *P*_*r*_, referred to as *P*_*t*_°99 and *P*_*r*_°99, respectively, are equal to 1.18 and 4.25 mm hr^−1^, respectively. All cases corresponding to *P*_*t*_ ≥ *P*_*t*_°99 (or *P*_*r*_ ≥ *P*_*r*_°99) are considered as extremes of domain‐scale precipitation (or local‐scale precipitation), and they are referred to as *P*_*t*_99 and *P*_*r*_99, respectively.

**Figure 2:**
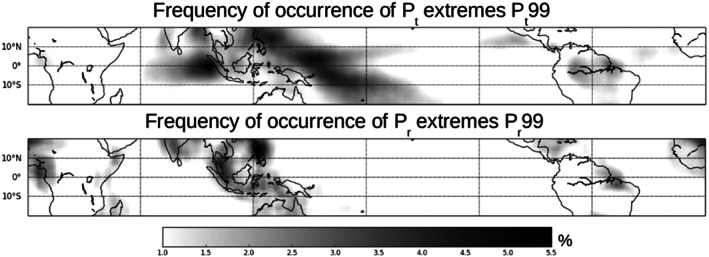
Frequency of occurrence of extreme events in (a) domain‐averaged precipitation (*P*_*t*_99) and (b) local‐scale precipitation (*P*_*r*_99) within the 10° × 10° domains of the tropics.

The frequency of occurrence of extreme precipitation events within each domain of 10° × 10° over the 1998 to 2010 period is shown in Figures 2a and 2b for *P*_*t*_99 and *P*_*r*_99, respectively. The frequency of *P*_*t*_99 extremes exceeds 1% over 40% of the tropical oceans and over 27% of tropical land. Most of the *P*_*t*_99 events occur over the warm pools of the tropical western Pacific and Indian oceans (Figure 2a). On the other hand, the frequency of *P*_*r*_99 events exceeds 1% over 43% of land and 21% of ocean regions in the tropics (Figure 2b).

### 2.3 Number of Convective Centroids, *N*

To characterize the spatial distribution of deep convection within 10° × 10° domains, we use 3‐hourly inter‐calibrated and gridded infrared brightness temperature (*T*_*b*_) data derived from geostationary satellites (GridSat‐B1 dataset, Knapp et al., 2011). The spatial resolution of this dataset is 0.07° × 0.07°. The GridSat temporal uncertainty is less than 0.1 K decade^−1^, and calibration uncertainty is less than 0.5 K for each satellite.

For each 3‐hourly snapshot of the *T*_*b*_ distribution within 10° × 10°, we define deep convective centroids as the points of local minima of the *T*_*b*_ field. Those points are detected in three steps. First, the *T*_*b*_ field is smoothed (to remove isolated convective pixels) by applying an exponential‐distance decay over a 0.7° × 0.7° area (10 × 10 GridSat pixels). Then, every time the smoothed *T*_*b*_ value at one particular pixel corresponds to the lowest value of the 3 × 3 pixels area centered around it, this pixel is considered as a local minimum of *T*_*b*_. If this local minimum is lower than 240 K, it is considered as a deep convective centroid (Figure 1c). The total number of convective centroids over a given domain is referred to as *N*.

This method differs from that used in many previous studies of convective aggregation (e.g., Stein et al., 2017; Tobin et al., 2012; Tompkins & Semie, 2017): those studies were based on the detection of deep convective clusters (defined as the ensemble of deep convective pixels sharing a common side), one convective cluster being associated with only one centroid whatever the size of the cluster (Figures S1a–S1c of the supporting information). The local minimum method used here can detect several convective centroids within a given cluster, and thus detects far more convective centroids than the clustering method. This allows for a more reliable determination of the convective organization index (the *I*_org_ metrics that requires at least 20 centroids to provide a reliable information).

### 2.4 Convective Organization Index: *I*_org_

Once all the convective centroids are identified within a given domain, the distances among nearest‐neighbor (NN) centroids are calculated. Then we characterize the spatial distribution of deep convective centroids by using the *I*_org_ index, which was proposed to quantify the degree of convective organization in cloud‐resolving simulations (Tompkins & Semie, 2017). Following Weger et al. (1992), the index compares the cumulative density function of the calculated NN distances (NNCDF) with that expected for a random distribution of the same number of convective centroids (see Figure S2 in the supporting information). For a random distribution associated with a Poisson process, the cumulative density function (PNNCDF) is given by a Weibull function (Stoyan et al., 1987) (equation (2)):

(2) $$PNNCDF=1-\exp(-\lambda \pi r^{2}).$$

where *λ* is the number of convective centroids per unit area, *r* is the nearest‐neighbor distance. Values of *I*_org_ larger (smaller) than 0.5 correspond to a clustered (regular) distribution of deep convective entities, while *I*_org_=0.5 corresponds to a randomly distributed convection.

Both *N* and *I*_org_ are calculated for each 10° × 10° domain of 20°S to 20°N, every 3 hr for the period of 1998 to 2010. The domains for which *N* is smaller than 20 are excluded from the statistics to remove cases which are close to clear‐sky conditions and to allow for reliable *I*_org_ estimates. Applying this rule reduces further the number of 10° × 10° domains considered in the analysis by 31% (from 397,268,984 to 272,585,649).

## 3 Link Between Precipitation Extremes and Convective Organization

**Figure 3:**
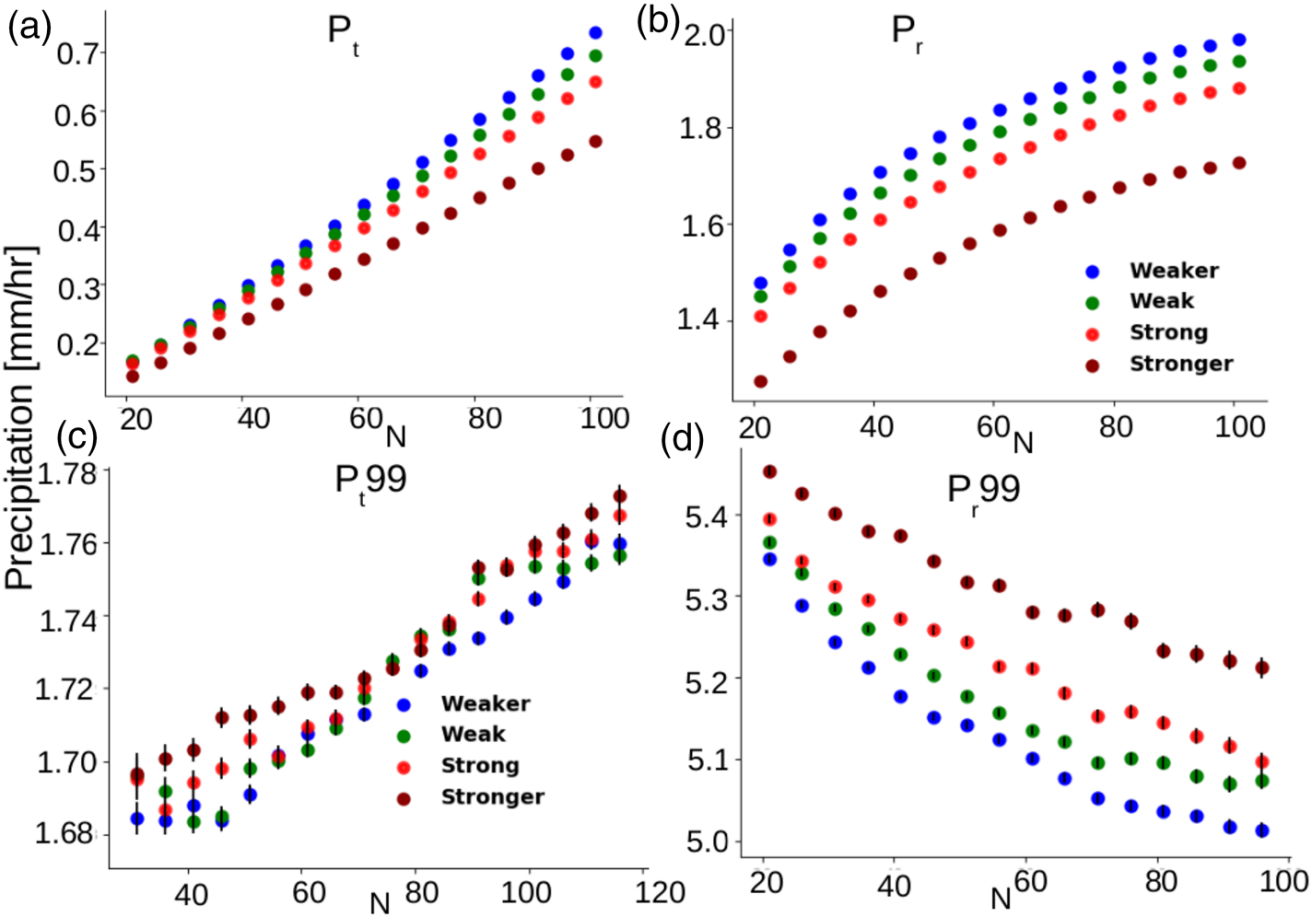
Relationship between *N* and (a) *P*_*t*_, (b) *P*_*r*_, (c) *P*_*t*_99, or (d) *P*_*r*_99 stratified, for each *N*, into quartiles of convective organization (*I*_org_). On (c) and (d), vertical bars indicate the error on the mean.

The relationships between precipitation, precipitation extremes, the number of convective centroids (*N*) and the degree of mesoscale organization (*I*_org_) within 10° × 10° domains are presented in Figure 3.

The relationships exhibit clear dependences of precipitation on both *N* and *I*_org_: *P*_*t*_ and *P*_*r*_ both increase with *N* (Figures 3a and 3b, Table S1). The increase of *P*_*t*_ is partly explained by the increase of the precipitating area (*A*_*r*_, which varies from 15% to 40% as *N* increases, see Figure S4 of the supporting information), and by the increase of the portion of this area that is covered by heavy rain (*a*_*s*_, which represents about 30% of the precipitating area). However, a simple scaling analysis based on equation (1) shows that the increase of *P*_*t*_ with *N* is mostly due to the increase of *A*_*r*_ ( $P_{r}\frac{\partial A_{r}}{\partial N}$ is 4 times larger than $A_{r}\frac{\partial P_{r}}{\partial N}$). Note that because of the dominance of the non‐precipitating area in 10° × 10° domains, *P*_*t*_ is much smaller than *P*_*r*_, sometimes by an order of magnitude. We also note that the precipitation intensity of *P*_*r*_ saturates for large *N* (Figure 3b) while *A*_*r*_ does not (see Figure S3a of the supporting information).

We now consider the influence on these relationships of the spatial organization of convection, as characterized by *I*_org_. At first order, *I*_org_ is anti‐correlated with *N*, a stronger clustering of convection being associated with fewer convective systems (supporting information, Figure S3). However, as illustrated in Figure S2, a given *N* (and at first order, a given precipitating area *A*_*r*_) can correspond to vastly different organizations of convection within the domain.

To investigate the influence of convective clustering on precipitation, for each N we stratify the relationships by quartiles of *I*_*org*_. Figures 3a‐b reveal that for a given N, a stronger clustering is associated with a weaker mean precipitation, both on the scale of the domain (*P*_*t*_) and at the scale of the precipitating area (*P*_*r*_). 75% of the decrease of *P*_*t*_ with increasing clustering is due to the shrinking of the precipitating area *A*_*r*_ and 25% due to a weakening of the local precipitation intensity associated with a decreased proportion of heavy precipitation within this area (*a*_*s*_, Figure S4, Table S1).

**Figure 4:**
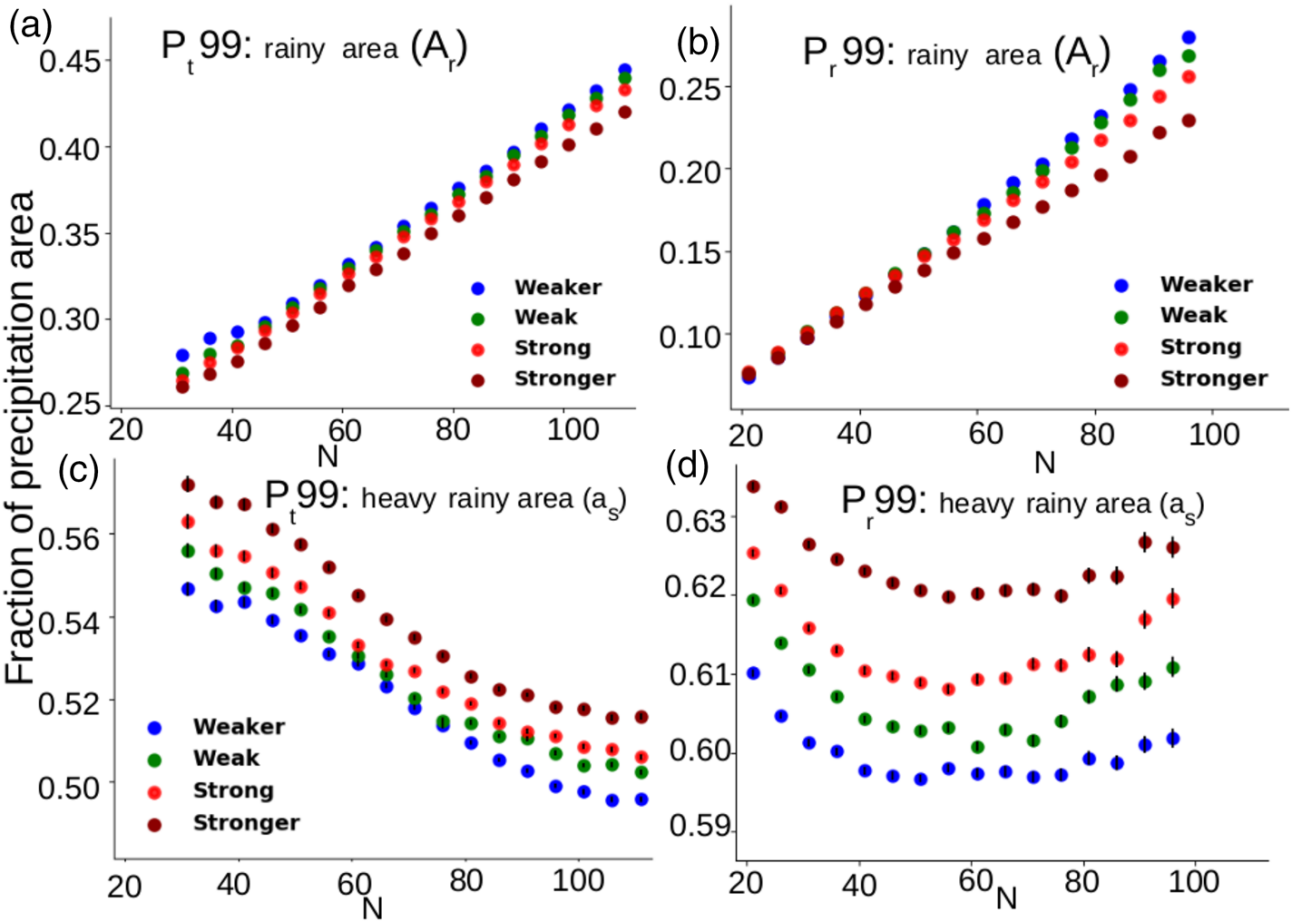
Relationship between *N* and the fractional area of precipitation extremes, stratified for each *N* into quartiles of *I*_org_: (a) the area covered by *P*_*t*_99 (*A*_*r*_), (b) the area covered by *P*_*r*_99 (*A*_*r*_), (c) the fraction of area covered by heavy rainfall (*a*_*s*_= $\frac{A_{s}}{A_{r}}$) of *P*_*t*_99, and (d) the fraction of area covered by heavy rainfall (*a*_*s*_= $\frac{A_{s}}{A_{r}}$) of *P*_*r*_99. On (c) and (d), vertical bars indicate the error on the mean.

Results for extreme precipitation are strikingly different from those for mean precipitation (Figures 3c and 3d). For a given *I*_org_, *P*_*t*_99 slightly increases with *N* while *P*_*r*_99 weakens (supporting information, Figures S3c and S3d, Table S1). Moreover, for a given *N* there is no systematic relationship between *P*_*t*_99 and *I*_org_, while *P*_*r*_99 increases systematically with the degree of convective clustering (Figures 3c and 3d). The rate of increase in *P*_*r*_99 with *I*_org_ is much higher than that of *P*_*t*_99 (Table S1). This behavior does not stem from a change in the precipitating area (Figure 4b) but from a systematic increase with the organization of the fractional area *a*_*s*_ covered by heavy precipitation (Figure 4d). It is found both over land and ocean regions (supporting informatio, Figure S5).

Note that the fractional area covered by heavy precipitation increases with *I*_org_ in all the 10° × 10° domains where extreme events occur (Figures 4c and 4d). The reason why it does not lead to an intensification of precipitation extremes at the domain‐scale (*P*_*t*_99) is because the large nonprecipitating area (*A*_*n*_=1−*A*_*r*_) has a stronger weight in *P*_*t*_ than the small portion of the domain covered by intensive rain rate.

## 4 Conclusion and Discussion

This study analyzes how mean precipitation and extreme precipitation relate to the number of deep convective entities (*N*) and to the spatial organization of deep convection (*I*_org_) at the scale of 10° × 10° domains in the tropics.

It shows that the domain‐averaged precipitation primarily increases with the number of deep convective entities within the domain, and that it does not depend much on convective organization. This is because the influence of convective organization on precipitating areas is largely damped by the presence of the non‐precipitating area.

Although the mean local precipitation increases as N increases or *I*_org_ decreases, the opposite is found for the intensity of precipitation extremes: *P*_*r*_99 intensifies as *I*_org_ increases or *N* decreases. The behaviors of mean precipitation and extreme precipitation with convective organization are thus strikingly different.

Extremes in domain‐scale precipitation (*P*_*t*_99), which occur mostly over the ocean warm pools (Figure 2a), primarily depend on the total number of convective centroids within the domain and are thus closely related to the total precipitating area, with little dependence on convective organization (Figure 4a). In contrast, extremes in local precipitation (*P*_*r*_99), which occur mostly over land (Figure 2b), mostly depend on the area of heavy precipitation, which itself primarily depends on the degree of convective clustering (Figure 4d).

How consistent are these observations with modeling results?

We show that at the scale of 10° × 10° domains, the spatial organization of deep convection (*I*_org_) is inversely related to the number of convective centroids (*N*), which is itself closely related to the total area of the domain covered by deep convection. This is consistent with idealized radiative‐convective equilibrium simulations showing that the area covered by deep convective clouds is reduced when the mesoscale organization of convection increases (Bretherton et al., 2005; Wing & Emanuel, 2014).

Regarding extreme precipitation, our observation that extremes in the domain‐averaged precipitation does not depend strongly on convective organization (Figure 3c) is consistent with the modeling results of Bao and Sherwood (2019). However, a close examination of the data reveals an increase of the fractional area of heavy precipitation as the degree of convective organization increases (Figure 4c). It means that the areas of intense precipitation associated with spatially organized convection are not large enough to influence the domain‐scale extreme precipitation *P*_*t*_99. On the other hand, we find strong observational evidence for an enhancement of extremes in local rainfall (*P*_*r*_99) when convective organization is stronger (Figure 3d), which is consistent with Singleton and Toumi (2013) and Pendergrass et al. (2016). In this case, precipitation is dominated by heavy rainfall, all the more that convection is organized (Figure 4d). This enhancement of local precipitation with convective organization would be consistent with an increase of the precipitation efficiency of convective systems in a moister environment (Bao & Sherwood, 2019).

Many studies indicate that extreme precipitation can cause huge threats to the economy, agriculture, infrastructure, and human lives (Spekkers et al., 2017; Wang et al., 2013). Observations suggest a strong link between the intensity of extreme rainfall at the local scale and the organization of deep convection, especially over land. Our study emphasizes therefore the need to understand the mechanisms that control convective organization, so that it will be possible to guide improvements of their representation in models, and eventually improve the model predictions of extreme precipitation.

## Supporting information



Supporting Information S1
