# Supplementary material for: Relationship Between Precipitation Extremes and Convective Organization Inferred From Satellite Observations
Source: Geophys Res Lett. 2020 May 6;47(9):e2019GL086927. doi: 10.1029/2019GL086927 (PMC7380318; doi:10.1029/2019GL086927)
Supplement: Supplementary file 1 — Supporting Information S1 [file GRL-47-e2019GL086927-s001.pdf]

Relationship between Precipitation Extremes and Convective Organization Inferred from Satellite Observations

Addisu Semie[1,2], Sandrine Bony [1]

[1]Laboratoire de Meteorologie Dynamique (LMD/IPSL), Sorbonne University, CNRS, Paris, France

[2]Computational Data Science Program, Addis Ababa University, Addis Ababa, Ethiopia

Contents of this file

1. Table S1

2. Figures S1 to S5

Introduction In this Supporting information, we provide a table that summarizes the rate of changes of precipitation intensity and precipitation fractional area with respect to N and I_{org} (Table S1). We also provide a snapshot of GridSat infrared brightness temperature data over 10° x 10° domain and we use it to illustrate how deep convective centroids are identified either through the clustering method or through the local minimum method (Figure S1). Examples of instantaneous satellite images and their corresponding cumulative density function of the nearest neighbor distance and their convective organization index are displayed in (Figure S2). The aggregated domain mean and extreme precipitation partitioned into I_{org}-N space (Figure S3). For each number of convective centroids (N), the fractional areas of precipitating region A_r and of heavy precipitating region a_s are stratified into quartiles of I_{org} (Figure S4). The intensity of extreme precipitation separately calculated over land and ocean (Figure S5).

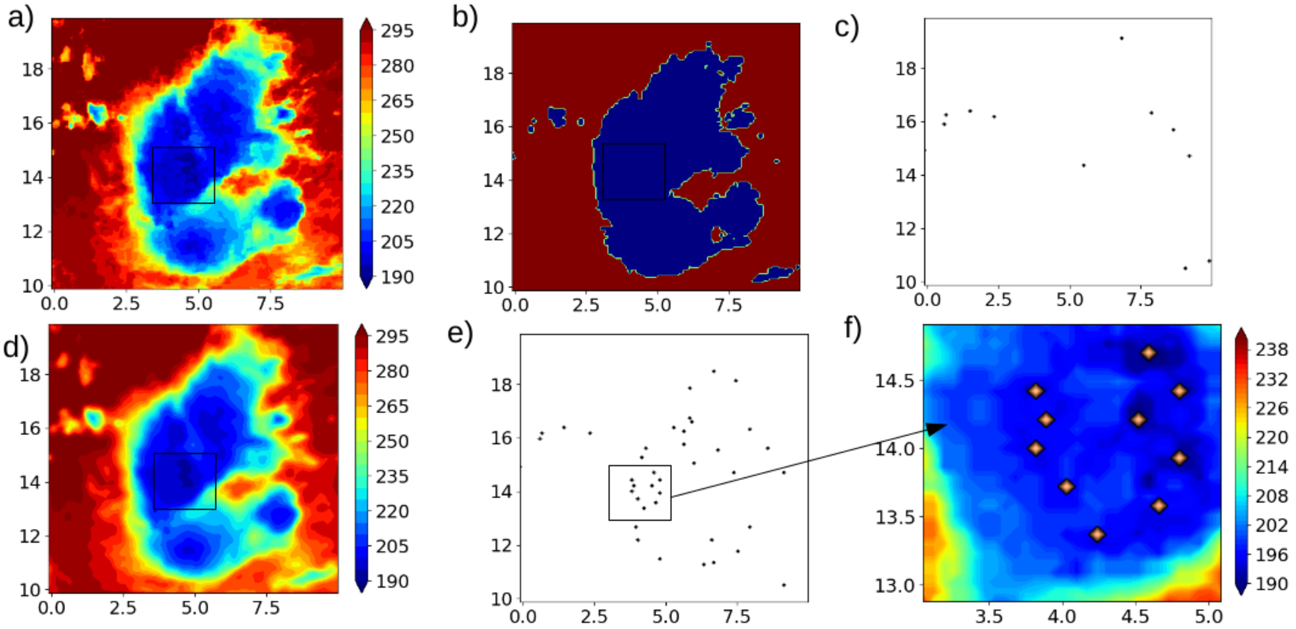

Figure S1. a) Snapshot of GridSat infrared brightness temperature data over a 10° x 10° domain (10° - 20^oN, 0° - 10^oE) at 1800 UTC on 21 Aug 1992. In the clustering method, all pixels associated with Tb $<$ 240 K are identified as "convective" (in blue on panel b) and convective pixels sharing a common side are considered as being part of the same cluster; each cluster is then associated with a single convective centroid (panel c). In the local minimum approach, the Tb field is first spatially smoothed using an exponential-distance decay (panel d). Then, local minima of this field are identified wherever the Tb value of a given pixel corresponds to the lowest value of the 3x3 adjacent pixels around it. Deep convective centroids are defined as those local minima where the smoothed Tb value (shown on panel d) is lower than 240 K (panel e). f) Zoom of panel (a) over a 2° x 2° area, showing the convective centroids identified through the local minimum approach.

Table S1. Rate of change with N or I_{org} of the precipitation fractional area and of the precipitation intensity (slopes of simple linear regression).

	δP_t	δP_r	δA_r	δa_s	$\delta P_t 99$	$\delta P_r 99$	$\delta A_r 99$	$\delta a_s 99$
δN	5.97x10^{-3}	5.44x10^{-3}	2.75x10^{-3}	1.18x10^{-3}	8.47x10^{-4}	-1.75x10^{-2}	2.45x10^{-3}	-0.61x10^{-3}
δI_{org}	-1.41	-1.8	-0.62	-0.35	-0.21	6.42	-0.81	0.25

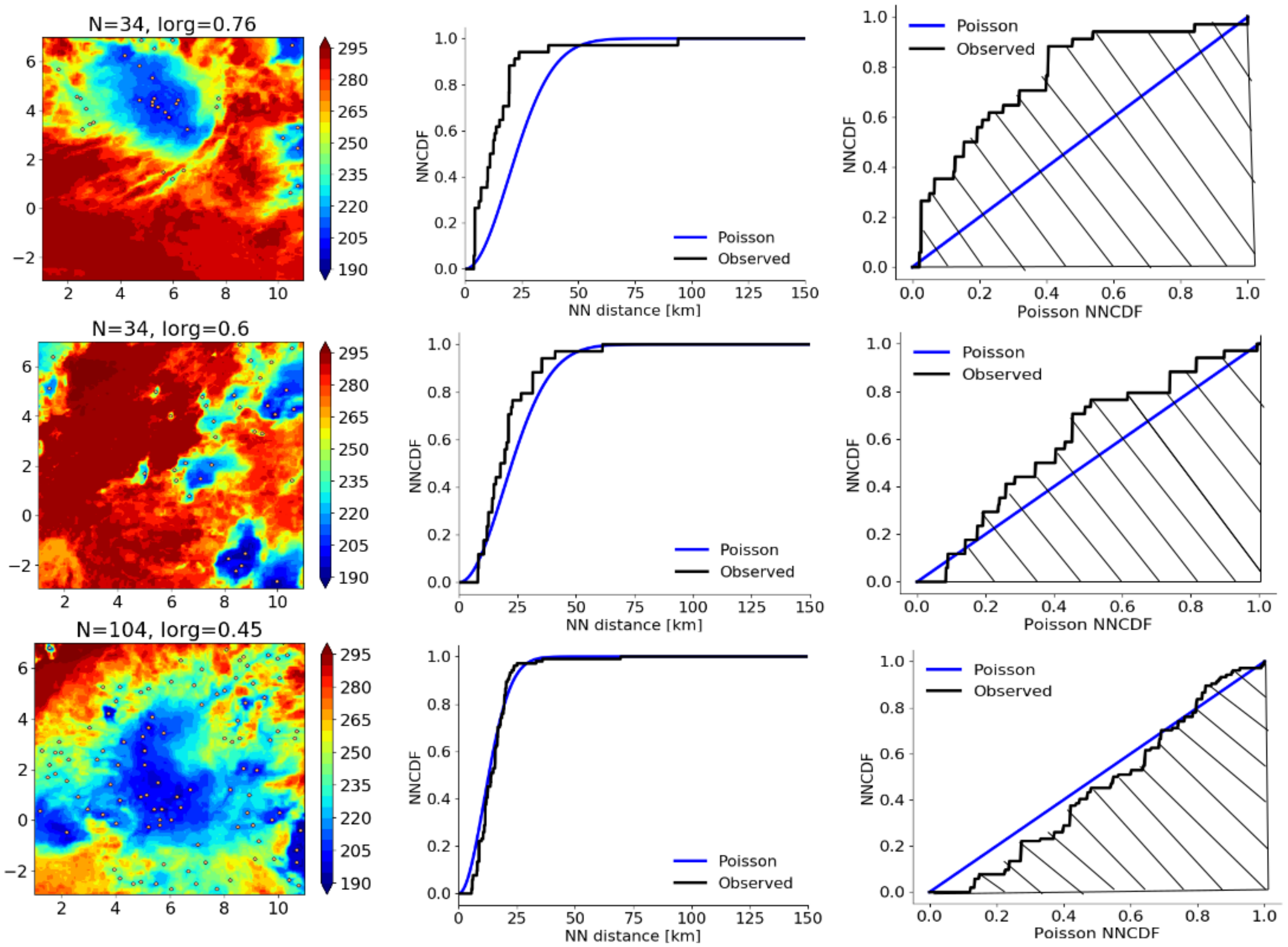

Figure S2. The first column shows snapshots of GridSat infrared brightness temperature data over 10° x 10° domains. The first two Snapshots display variations in the spatial distribution of deep convection for a given number of convective centroids (N=34). The last snapshot in this column shows an example of more regular distribution for a high number of deep convective centroids. The second column displays the corresponding NNCDF versus nearest neighbor distance of observed and idealized Poisson convective distribution. The third column indicates the corresponding NNCDF versus Poisson NNCDF diagram. I_{org} corresponds to the shaded area under the curve.

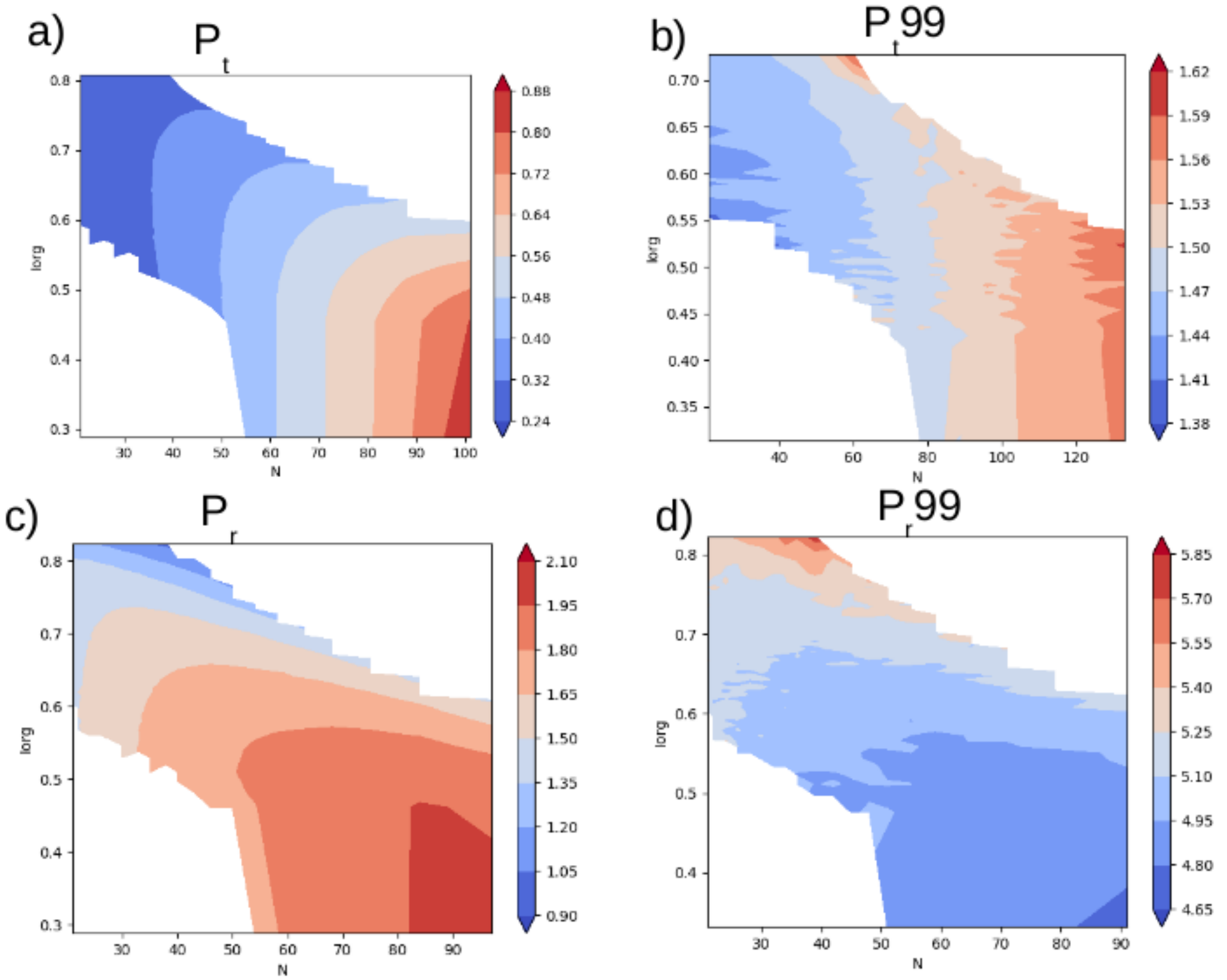

Figure S3. Precipitation diagnostics a) P_t b) P_t99 c) P_r d) P_r99, aggregated in space (20^oS - 20^oN) and in time (1998-2010) are binned by I_{org} and N. All the mean values of the diagnostics falling within I_{org}-N bins associated with a number of points lower than 0.01% of the total number of mesoscale domains are masked out (white areas).

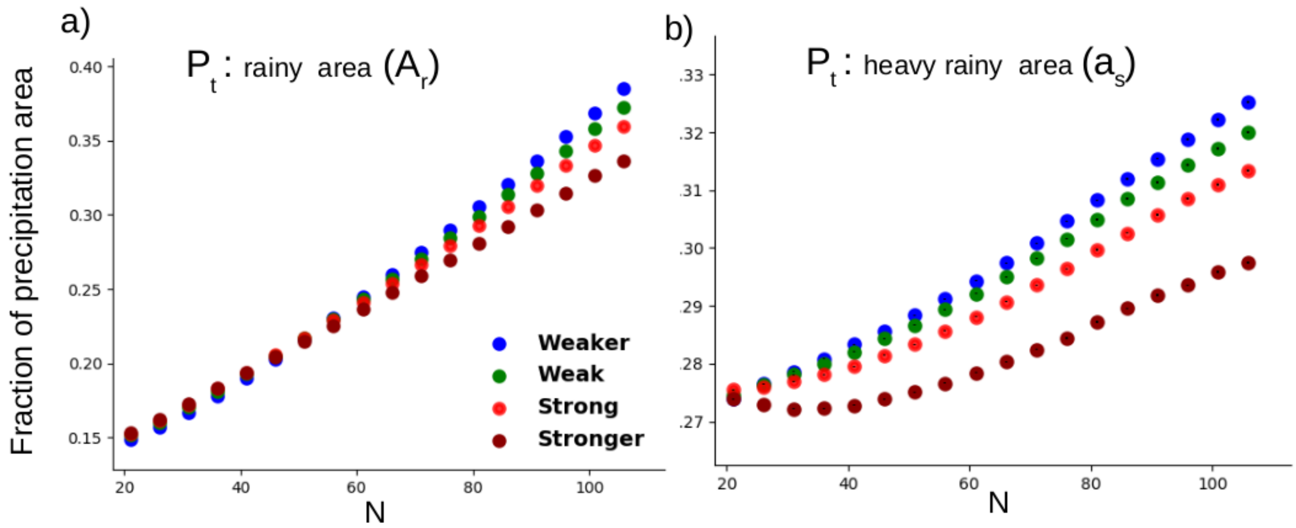

Figure S4. For each N, stratification into I_{org} quartiles of a) the A_r fractional area of precipitation and of b) the ratio of heavy rainfall ($a_s = \frac{A_s}{A_r}$). The spatial scale of P_r is as large as A_r. This implies the spatial scale of P_r is as small as 15% to 40% of the spatial scale of P_t (10° x 10°).

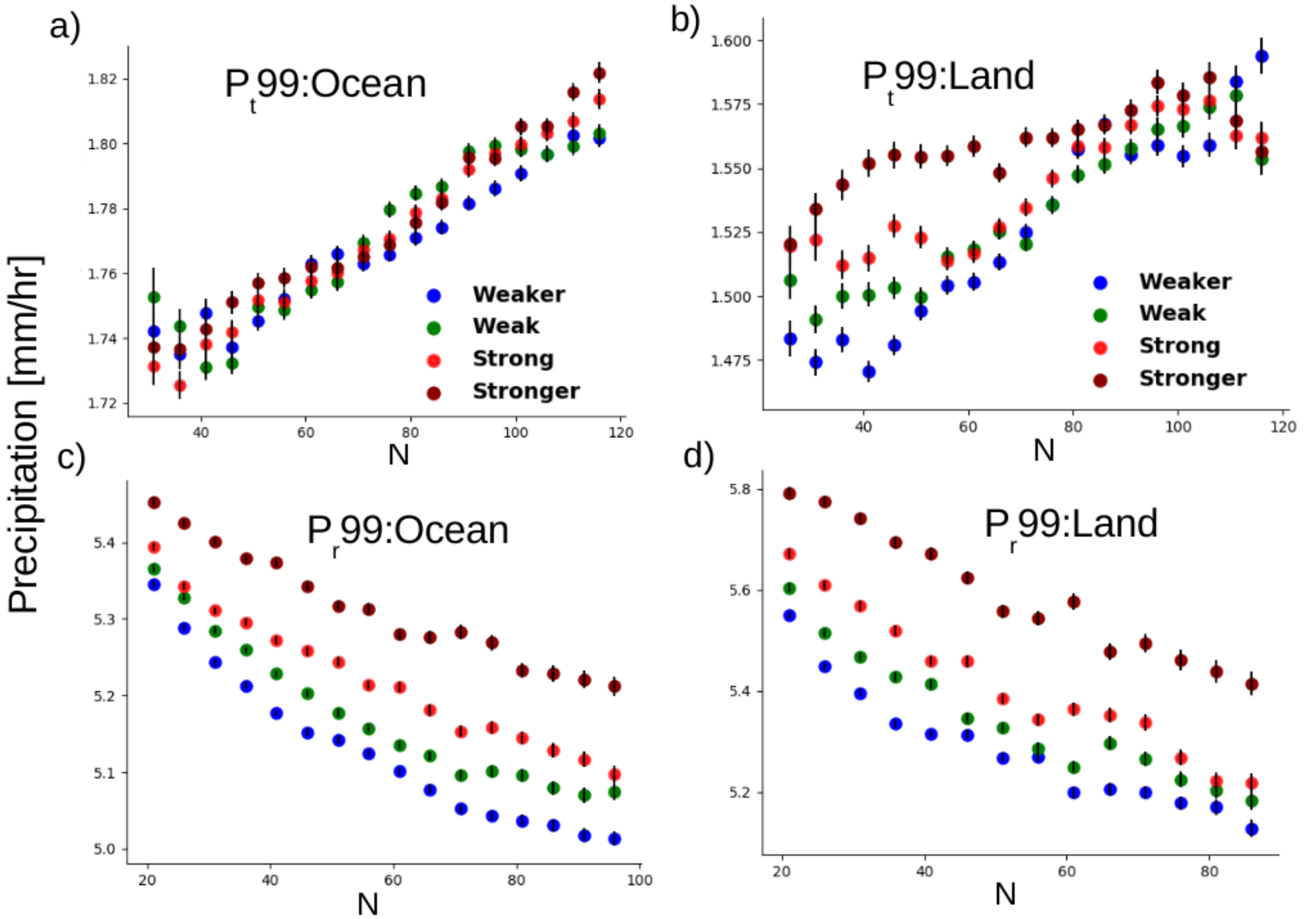

Figure S5. Relationship between N and precipitation extremes, stratified for each N into quartiles of I_{org}: a) P_t99 over tropical oceans, b) P_t99 over tropical land, c) P_r99 over tropical oceans and d) P_r99 over tropical land. Vertical bars indicate the error on the mean.
